# Sensorimotor performance and haptic support in simulated weightlessness

**DOI:** 10.1007/s00221-020-05898-5

**Published:** 2020-08-07

**Authors:** Bernhard Weber, Michael Panzirsch, Freek Stulp, Stefan Schneider

**Affiliations:** 1 German Aerospace Center, Institute of Robotics and Mechatronics, Oberpfaffenhofen, Germany; 2 Institute of Movement and Neurosciences, German Sport University, Cologne, Germany

**Keywords:** Aerospace simulation, Force feedback, Haptic interfaces, Sensorimotor performance, Weightlessness, Water immersion

## Abstract

The success of many space missions critically depends on human capabilities and performance. Yet, it is known that sensorimotor performance is degraded under conditions of weightlessness. Therefore, astronauts prepare for their missions in simulated weightlessness under water. In the present study, we investigated sensorimotor performance in simulated weightlessness (induced by shallow water immersion) and whether performance can be improved by choosing appropriate haptic settings of the human–machine interface (e.g., motion damping). Twenty-two participants performed basic aiming and tracking tasks with a force feedback joystick under water and on land and with different haptic settings of the joystick (no haptics, three spring stiffnesses, and two motion dampings). While higher resistive forces should be avoided for rapid aiming tasks in simulated weightlessness, tracking performance is best with higher motions damping in both land and water setups, although the performance losses due to water immersion cannot be compensated. The overall result pattern also provides insights into the causal mechanism behind the slowing effect during aiming motions and decreased accuracy of tracking motions in simulated weightlessness. Findings provide evidence that distorted proprioception due to altered muscle spindle activity seemingly is the main trigger of impaired sensorimotor performance in simulated weightlessness.

## Introduction

During their space missions, astronauts have to perform delicate and demanding tasks reliably and precisely, under the adverse conditions of weightlessness. Prior research, however, repeatedly documented that human motor performance in weightlessness is degraded under certain conditions  (Lackner and DiZio 2000; Manzey 2017). Impairments have been found across different elementary free motion tasks (e.g., aiming and tracking in dual-task paradigms) and force regulation (e.g., production of finely graded forces), which are most evident in the initial phase of exposition to weightlessness (Hermsdörfer et al. 1999, 2000; Kanas and Manzey 2008).

Three main explanations have been proposed for these sensorimotor impairments in weightlessness: (1) distorted proprioception due to altered muscle spindle activity, e.g., Bock (1998), (2) attentional deficits due to the general workload of space missions, e.g., Manzey et al. (2000), and (3) altered motor control strategy to avoid instability of the weightless body during limb movements, e.g., Mechtcheriakov et al. (2002).

In terrestrial (tele-)operation systems where, e.g., medical instruments, aircraft, or robots are controlled, the operator is usually supported by various forms of haptic support. Joysticks, for instance, usually have a spring mechanism to stabilize deflections and support re-centering. Specifically, the spring stiffness of a joystick is a haptic augmentation of position (higher resistance equals larger deflection) and viscous damping a haptic augmentation of velocity (higher velocity results in higher damping). Studies showed that moderate values of stiffness improve tracking as well as precision aiming performance  (Jones and Hunter 1990; Weber and Rothkirch 2014). Damping helps performing smoother movements and attenuates unintended movement impulses during tracking (Jones and Hunter 1993; Lange 2014) and adequate damping even supports rapid aiming motions (Crommentuijn and Hermes 2011).

Here, the intriguing question arises whether, and to which extent, these haptic settings of a human–machine interface (HMI) are still beneficial in weightlessness or whether these settings have to be adjusted to be beneficial. Answering these questions will also shed light on the assumed causal mechanisms underlying impaired sensorimotor performance, since these haptic settings might moderate the effects of weightlessness.

These questions are investigated by comparing aiming and tracking task performance on land and in weightlessness, simulated by neutral buoyancy during shallow water immersion. Astronauts also complete an intensive training schedule to familiarize with weightlessness effects simulated by water immersion (Bolender et al. 2006). The discussed main mechanisms leading to performance losses in microgravity should also be relevant for simulated weightlessness during water immersion. Although the lack of gravito-inertial force which potentially affects the vestibular system during microgravity is not given by water immersion, very similar effects during shallow water immersion and spaceflight have been reported for aiming (Weber et al. 2018), tracking (Weber et al. 2016), and isometric force production tasks (Dalecki 2013).

From a practical perspective, the main contribution of the current work is the determination of preferable haptic settings for sensorimotor performance in simulated weightlessness compared to land conditions. From a more theoretical perspective, the variation of haptic settings could moderate potential performance losses and, thus, contribute to a better understanding of the underlying mechanisms of performance losses and help determining ways how to attenuate or even eliminate the sensorimotor deficits in simulated weightlessness using appropriate haptic settings.

## Underlying mechanisms of sensorimotor impairment and expected effect patterns in simulated weightlessness

**Table 1:** Potential mechanisms of impaired sensorimotor performance and expected effects in simulated weightlessness

Performance dimensions				
Motion planning		Affected		
Feedforward control/rapid motion	Less affected	Affected	Affected	Affected
Feedback control/slow motion	More affected			
Anisotropy	Dependent on # of moved limbs	None	Dependent on inertial load of moved limbs	Dependent on drag coefficient of moved limbs

### Distorted proprioception

Several researchers explained sensorimotor impairment in altered gravity conditions and under water by disturbed proprioception (Bock 1998; Bock et al. 1992; Dalecki et al. 2012b; Fisk et al. 1993; Manzey et al. 2000). According to this hypothesis, muscle spindle activity which is crucial for proprioception is altered by the weightlessness of the body and limbs (Lackner and DiZio 2000). During closed-loop tasks which are feedback-controlled tasks (such as slow and precise aiming or tracking), this effect should be particularly evident. The impact of weightlessness on open-loop or feedforward-controlled motion (such as rapid aiming), however, should be smaller (Fisk et al. 1993). Moreover, the effect of disturbed proprioception should be stronger, the more limbs are involved in movement, as reported in research on patients without proprioception due to large-fiber sensory neuropathies (Ghez et al. 1990).

### Attention deficit

Manzey et al. (1995, (2000) argued that the defective proprioception approach is not sufficient to explain the performance decrements which they found in later phases of a mission when proprioceptive adaptation should be completed (Kanas and Manzey 2008). They argued that sensorimotor performance losses in the latter phases were caused by decreased attentional resources due to general stressors (Bock et al. 2003; Fowler et al. 2008; Manzey et al. 1993, 1995). Performance losses due to attentional deficits mainly affect the feedforward-controlled part of motion (Fowler et al. 2000, 2008; Weber et al. 2019).

### Altered control strategy

An altered strategy of motor control when performing pointing arm movements in weightlessness was discussed by Berger et al. (1997) and Mechtcheriakov et al. (2002) as an additional explanatory approach. They argued that the slowing of arm motions reflects a strategy to avoid too high counter-forces in other body parts which are difficult to compensate in weightlessness. Consequently, the magnitude of these effects should increase with a higher inertial load of involved body parts and be higher for rapid and large amplitude movements (such as rapid aiming).

### Water viscosity

During water immersion, dynamic water viscosity effects have to be taken into account (Brown 1961). Motions are slowed down and the magnitude of this effect is linearly related to motion velocity (Kerr 1973, 1978). Potential effects of water viscosity should mainly emerge during rapid motions and the effect should be moderated by the respective drag of the moved body part.

Table 1 summarizes the expected effect patterns of the proposed mechanisms for sensorimotor deficits in simulated weightlessness. Moreover, it is indicated whether effect sizes are expected to be dependent on motion direction (anisotropy). In the present study, motion planning, feedforward-controlled rapid motion, as well as feedback-controlled precision matching were explored during an aiming task. Feedback-controlled continuous, slow motions were also explored during a tracking task.

## Haptic settings and simulated weightlessness effects

In the current study, we focus on the haptic settings at the input device as a potential moderator variable, allowing a deeper insight in the causal mechanism of impaired performance in simulated weightlessness.

### Distorted proprioception and haptics

Providing haptics information about limb position (i.e., stiffness) and velocity (i.e., damping) might, on one hand, help to compensate impaired proprioception in weightlessness (Bringoux et al. 2012). Then, feedback-controlled motions should be less affected. On the other hand, it is well conceivable that the opposite is true, since there are studies reporting reduced precision of force production in simulated weightlessness, which has been explained by distorted proprioception (e.g., Dalecki et al. 2012b). Then, haptic settings should be less supportive or even detrimental during feedback-controlled motions in simulated weightlessness.

### Attention deficit and haptics

Analogously, the question whether attentional demands required for performing motions in simulated weightlessness increase or decrease when enabling haptic settings might depend on how adequately these settings support task execution.

### Altered control strategy and haptics

If the changed control strategy approach holds true, the beneficial effects might also depend on the intensity of the haptic variables: the stabilizing nature of moderate stiffness and damping could have a positive effect during rapid motions in simulated weightlessness, since motion dynamic is reduced by these haptic settings. When applying inadequately high stiffness and damping, however, body stabilization might even be more difficult in simulated weightlessness as compared to conditions without any haptic settings (Weber et al. 2019), potentially leading to additional slowing.

Thus, the effect direction of additional haptic settings in simulated weightlessness is hard to predict and might be contingent on the intensity of these effects. Consequently, the effects of the haptic setting will be explored as an open research question in the following experiment.

## Method

### Apparatus

**Fig. 1:**
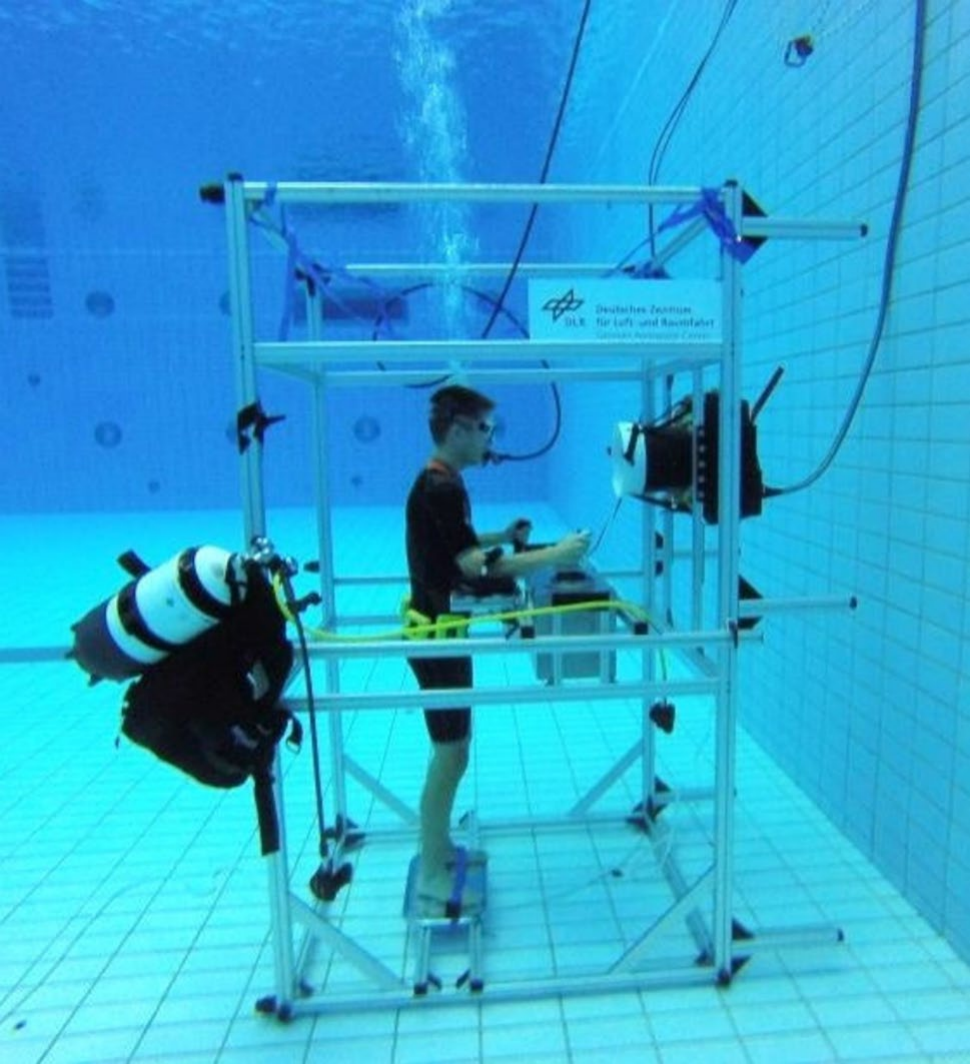
Experimental setup in water condition

A 230 cm $\times$ 110 cm $\times$ 110 cm aluminum frame was used for the water as well as for the land conditions (see Fig. 1), i.e., the identical setup was placed on deck or on the bottom of the pool, respectively. A 15$^{\prime \prime }$ monitor (with a cylindrical hood to avoid reflections on the screen) was mounted at the rear side of the frame and the joystick module was inserted between two rails in the center of the frame.

A force feedback joystick (the DLR SENSO-Stick developed by SENSODRIVE) with two axes, each axis with a workspace of $\pm \, 20^{\circ }$ and a maximum torque of 2.01 Nm, was used for the current experiment. A 1:2 motion upscaling was implemented to minimize the potential effect of water viscosity. Targets could be reached with a deflection of $8^{\circ }$, i.e., 2 cm motion at the top of the joystick handle. The haptic properties of the joystick could be varied by adjusting the torques generated by the two brushless DC motors (torque resolution 0.03 Nm). Thus, the desired spring stiffness and damping effects could be produced. When deactivating the motors, free and unrestricted motion (isotonic configuration) is possible due to the good backdrivability of the device. A flexible latex gaiter connected joystick module and the lower end of the handle. A pressure regulator was connected to the module ensuring that the environmental pressure and the pressure in the joystick module were identical to avoid the flexible gaiter to deform under water. At the joystick module, a padded armrest with a padded elbow strap was attached. An additional handle for the left hand was installed at the same rack as the joystick.

The complete experiment was conducted in the Aquatics Center of the German Sports University in Cologne. In the water conditions, the setup was immersed in a 20 $\times$ 20 m pool with 5 m depth and a temperature of $27\,^{\circ }{\text {C}}$, with the frame standing on the bottom of the pool (i.e., joystick at 3.6 m depth). Air supply was provided by a hose connected to a bottle with compressed, normal air on deck. For reasons of safety, an additional SCUBA jacket with air supply was attached to the frame. In the land condition, the setup was located on the deck and curtains at each frame side were used to avoid visual distraction. In both conditions, the experiment was supervised from a control desk on deck. The experimental software ran on a laptop which was connected to a PC calculating the force signals for the joystick. Furthermore, data recording was performed on this laptop with a sampling rate of 50 Hz and it was connected to the 15$''$ monitor to display the experimental graphical user interface (GUI). There was a viewing distance of 70 cm from subject’s eyes to the display. Compared to the land condition, the GUI size was scaled down in the water condition to compensate the underwater magnification effect, caused by the light refraction at the goggles (refractive index of 1.33 in freshwater). Accordingly, the distance between aiming start and target positions in the GUI, e.g., was 6.5 cm in the land and 4.9 cm in the water condition.

### Experimental tasks

**Fig. 2:**
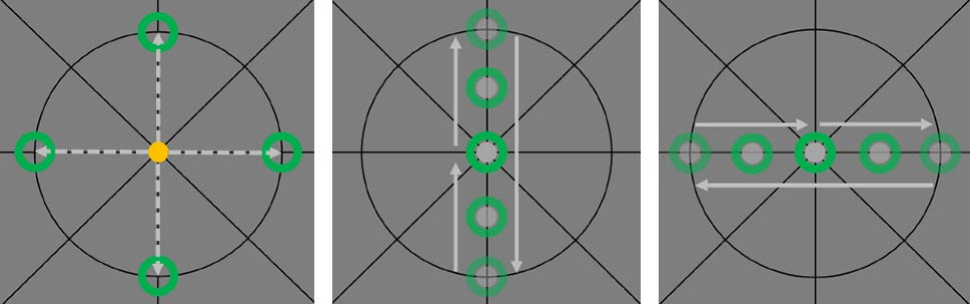
Experimental tasks. Left: the four aiming tasks with cursor (yellow) and target (green). Middle and right: vertical and horizontal tracking

The experiment consisted of a series of aiming and tracking subtasks performed with the force feedback joystick. For both subtasks, subjects had to move a black circular cursor to the starting point at the center of the crosshairs. Upon reaching the center, the cursor color turned from black to green and a countdown appeared. When the starting position has been held for 2 s, the countdown disappeared, and the cursor color turned either yellow (aiming task) or gray (tracking task) (see Fig. 2).

#### Aiming

Subjects were instructed to perform the respective aiming task immediately. During aiming, an upper, lower, left, and right target ring (random order) had to be matched (see Fig. 2, left). Subjects were asked to match the targets “as quickly as possible”, to trigger rapid, feedforward-controlled motions. When entering the desired end position in the inner of the ring (0.5 mm threshold), the cursor color changed to green and after having held this position for 0.5 s. Hence, subjects had to move very precisely to match the target, i.e., feedback-based corrections were required to solve the task. When the task was successfully completed, subjects went back to the starting position to continue with the next trial.

#### Tracking

During tracking, the cursor color switched to gray after the countdown. The target ring automatically started moving along the vertical or horizontal axes (random order) with constant speed (13 mm/s). The comparably slow target motion mainly required feedback-controlled corrections of the cursor’s position and subjects were accordingly asked to “match the target as precisely as possible”. For both directions, targets moved from start to the two intersections of axis and circle and returned to start (see Fig. 2, middle and right).

In each experimental trial, subjects always completed the aiming tasks first (i.e., four aiming movements with the different target positions) and then proceeded with the tracking task (i.e., two tracking subtasks with the different motion directions, each lasting 20 s). One complete trial lasted about 1.5 min.

### Experimental design

The two independent variables were Setup (water vs. land) and Haptic Setting of the force feedback joystick. An isotonic (no haptics), four stiffnesses (stiffness 1: 0.075 Nm/rad; stiffness 2: 0.225 Nm/rad; stiffness 3: .375 Nm/rad; stiffness 4: 0.525 Nm/rad), and two dampings (damping 1: 0.045 Nm s/rad; damping 2: 0.090 Nm s/rad) were selected based on findings of prior studies Lange (2014). Stiffnesses and dampings were tested separately and were not combined.

A 2 (Setup) $\times$ 7 (Haptic Settings) within-subject design was implemented, resulting in 14 experimental trials. Each setup session lasted about 20 min. The order of the Setup condition was counterbalanced across subjects. The Haptic Setting order was randomly chosen for each subject.

### Procedure

First, subjects were given instructions on the experimental task and procedure. In the water condition, subjects put on a 7 mm short sleeves neoprene suit to avoid hypothermia, goggles (prepared with anti-fog spray) and a belt with individually adjusted diving weights to achieve neutral buoyancy. Then, they descended, entered the frame from above, put their feet in a foot strap, attached the elbow strap, and started with the experiment. In the land condition, subjects entered the frame, put on hearing protectors (i.e., protective earmuffs with a padded headband), and started with the experiment. No googles were put on in this condition, because googles steamed up even more quickly due to the higher skin temperature. Both water and land conditions were completed on different days with a maximum interval of 8 days. Prior to the main trials, a training trial (two aiming and one tracking task) was started. In this training trial, the spring stiffness 2 and motion damping 2 were activated to familiarize subjects with the haptic effects. Subsequent to each experimental trial, subjects answered a question on perceived workload [“Please rate your overall workload during the last task”, adopted from Vidulich and Tsang (1987)].

### Sample and ethics approval

Twenty-two subjects (3f, 19m) with an average age of ${M} = 27.8$, ${\text {SD}} = 8.0$ years. participated in the study. Most of them had SCUBA diving experience [two beginners (five dives each), sixteen experienced divers ($>15$ dives)] except for four subjects (no or only one dive) who were given a theoretical instruction and practical diving training. Spectacle wearers were asked to wear contact lenses for the experiment. All participants signed a written informed consent and the study was approved by the Ethics Committee of the German Sports University.

### Performance measures and data analysis

During aiming, motion planning was measured by “Reaction Time” (RT = time from task start until exceeding a pre-defined threshold velocity of 21 mm/s). Rapid, feedforward-controlled motion by “Rapid Motion Time” (RMT = time [cursor center touches target ring] - RT) and feedback controlled, slow matching motions by “Fine Motion Time” during aiming (FMT = time from touching target ring until matching the middle of the target ring). During the feedback-controlled tracking task, a “Tracking Error” was calculated (ERROR = root-mean-square error (RMSE) of distance between cursor and target). Unit of measurement was millimeters, while 1 mm equals a visual angle of $0.082^\circ$ in our setup. Tracking error was measured from target motion onset until the target reached the end position.

All analyses followed the same scheme: a repeated-measures Direction (upper, lower, right, left target or horizontal vs. vertical tracking axis, respectively) $\times$Setup (water vs. land) $\times$Haptic Setting (isotonic, 4 stiffnesses, 2 dampings) ANOVA (rmANOVA) was conducted on each performance measure. Sphericity was tested (Mauchley’s sphericity test) and Greenhouse–Geisser or Huyn–Feldt corrections were made in case of non-sphericity. Subsequently, effect sizes (Hedges’ *g*) and post hoc contrasts with Bonferroni correction were performed (two-tailed testing for Haptic Setting comparisons, one-tailed for Setup comparisons, since performance losses were expected for the water compared to the land condition). For the following analyses, two subjects had to be excluded (interruptions due to problems with the diving mask in the first case and earache in the underwater condition in the second case).

## Results

### Aiming

#### RT

**Fig. 3:**
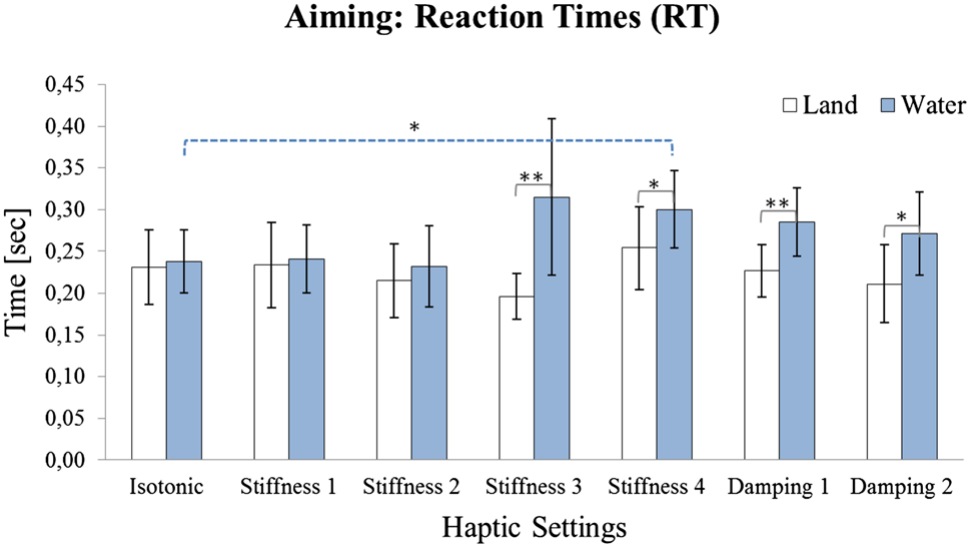
Mean aiming reaction times (s) for land (white) and water (blue) conditions with 95% confidence intervals; Significant differences are indicated by asterisk (*${p}<0.05$; **${p}<0.01$)

RmANOVA on the RTs revealed a significant Setup main effect ($$F \, (1, 19) = 6.37;$$$$p < 0.05$$) and a significant Setup $$\times$$Haptic Setting interaction effect ($$F \, (4.94, 93.91) = 2.98$$; $$p < 0.05$$; see Fig. 3 and Table 2 for detailed statistical results). RTs increased under water, but this effect was only significant for the higher stiffnesses 3 and 4 and both dampings as indicated by post hoc contrasts and small-to-moderate-effect sizes. No significant effect was evident in the isotonic condition ($$g = 0.07$$). Obviously, there was no general delay effect under water, but increased RTs in conditions with stronger haptic effects.

**Table 2:** Result overview for the AIMING experiment with reactions times (RT), rapid motion times (RMT), and fine motion times (FMT) (s)

${{{N} = 20}}$	Isoton.	Stiff. 1	Stiff. 2	Stiff. 3	Stiff. 4	Damp. 1	Damp. 2	rmANOVA results	
RT									
Land	0.231	0.234	0.215	0.196	0.254	0.227	0.211	Setup	$\varvec{p} <\mathbf{0.05 }$
	(0.102)	(0.101)	(0.101)	(0.061)	(0.113)	(0.071)	(0.106)	*F*(1, 19) = 6.37	($\eta$^2^ = 0.25)
*t*-Test (isot.-haptic)									
*g* (isot.-haptic)		0.03	0.15	0.41	0.21	0.04	0.19	**Setup** $\times$**Haptic**	$\varvec{p} <\mathbf{0.05 }$
Water	0.238	0.241	0.232	0.315	**0.300**	0.285	0.271	*F*(4.94, 93.91) = 2.98	($\eta$^2^ = 0.14)
	(0.086)	(0.092)	(0.110)	(0.215)	(0.106)	(0.094)	(0.114)	Huyn–Feldt	
*t*-Test (isot.-haptic)					${\varvec{p}} <\mathbf{ 0.05 }$				
*g* (isot.-haptic)		0.03	0.06	0.46	0.63	0.51	0.32		
*t*-Test (WL)				${\varvec{p}} <\mathbf{ 0.01 }$	${\varvec{p}} <\mathbf{ 0.05 }$	${\varvec{p}} <\mathbf{ 0.01 }$	${\varvec{p}} = \mathbf{0.01}$		
*g* (WL)	0.07	0.07	0.16	0.74	0.41	0.68	0.54		

RMT									
Land	0.453	0.538	0.530	**0.580**	**0.663**	0.479	**0.586**	**Setup:**	$\varvec{p} <\mathbf{0.05 }$
	(0.174)	(0.267)	(0.177)	(0.199)	(0.243)	(0.131)	(0.149)	F (1, 19)= 6.76	($\eta$^2^= 0.26)
*t*-Test (isot.-haptic)				${\varvec{p}} <\mathbf{0.001 }$	${\varvec{p}} <\mathbf{0.001 }$		${\varvec{p}} = \mathbf{0.01}$		
*g* (isot.-haptic)		0.37	0.43	0.67	0.97	0.17	0.80	**Haptic:**	$\varvec{p} <\mathbf{0.001 }$
Water	0.543	0.583	0.619	**0.735**	0.731	0.612	0.604	*F*(5.14, 97.59)= 9.06	($\eta$^2^ = 0.32)
	(0.183)	(0.253)	(0.297)	(0.312)	(0.369)	(0.252)	(0.206)	Huyn–Feldt	
*t*-Test (isot.-haptic)				${\varvec{p}} <\mathbf{0.05 }$					
*g* (isot.-haptic)		0.18	0.30	0.74	0.63	0.31	0.31		
*t*-Test (WL)	${\varvec{p}} <\mathbf{0.05 }$			${\varvec{p}} <\mathbf{0.01 }$		${\varvec{p}} <\mathbf{0.05 }$			
*g* (WL)	0.51	0.16	0.31	0.55	0.20	0.64	0.07		

FMT									
Land	1.695	1.695	1.944	1.774	1.752	1.864	1.735	**Setup:**	$\varvec{p} <\mathbf{0.01 }$
	(0.519)	(0.310)	(0.577)	(0.325)	(0.336)	(0.178)	(0.461)	*F* (1, 19)= 14.26	($\eta$^2^ = 0.43)
*t*-test (isot.-haptic)									
*g* (isot.-haptic)		0.00	0.44	0.18	0.13	0.43	0.08		
Water	2.224	2.041	2.643	2.799	2.379	2.490	2.038		
	(0.689)	(0.411)	(2.289)	(1.355)	(0.680)	(1.269)	(0.534)		
*t*-Test (isot.-haptic)									
*g* (isot.-haptic)		0.32	0.24	0.52	0.22	0.26	0.30		
*t*-Test (WL)	${\varvec{p}} <\mathbf{0.01 }$	${\varvec{p}} <\mathbf{0.01 }$	*p* = 0.059	${\varvec{p}} = \mathbf{0.001}$	${\varvec{p}} <\mathbf{0.001 }$	${\varvec{p}} <\mathbf{0.01 }$	${\varvec{p}} <\mathbf{0.05 }$		
*g* (WL)	0.85	0.93	0.41	1.02	1.15	0.65	0.60		

Means, standard deviations (in parentheses), significant results of rmANOVA (*p* values and partial ${{\eta }^{{2}}}$), effect sizes (Hedges’ *g*), and post hoc contrasts for water–land (WL) comparisons and post hoc contrasts for the haptic setting comparisons (isotonic vs. haptic condition; significant means shown in bold)

#### RMT

**Fig. 4:**
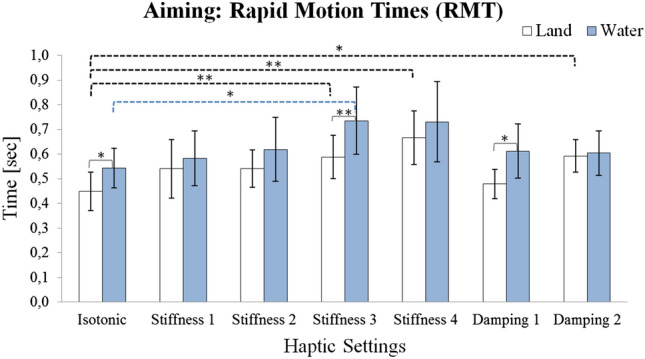
Mean rapid motion times (s) for land (white) and water (blue) conditions with 95% confidence intervals. Significant differences are indicated by asterisk (*$p <0.05$; **$p <0.01$)

Analyzing RMTs also showed a significant Setup main effect, [$F \, (1, 19) = 6.76$; $p < 0.05$], i.e., subjects required more time to perform rapid aiming motions under water ($g = 0.51$ in the isotonic condition), see Fig. 4. The other Setup contrasts were inconsistent and only reached significance in the stiffness 3 and damping 1 conditions. Moreover, a significant Haptic Setting main effect [$F \, (5.14, 97.59) = 9.06$; $p < 0.001$] reflected increased RMTs for certain haptic conditions. Contrasts with the isotonic conditions reached significance for stiffness 3 in both setups and additionally for stiffness 4 and damping 2 in the land condition.

#### FMT

**Fig. 5:**
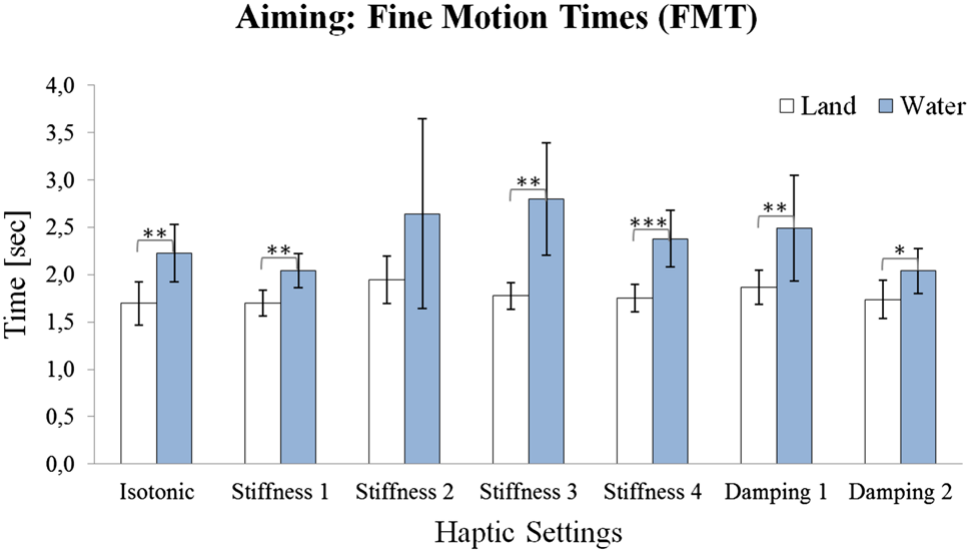
Mean aiming fine motion times (s) for land (white) and water (blue) conditions with 95% confidence intervals. Significant differences are indicated by asterisk (*$p <0.05$; **$p <0.01$; ***$p <0.001$)

Finally, rmANOVA on the FMTs also yielded a significant Setup main effect [$F \, (1, 19) = 14.26$; $p < 0.01$], i.e., FMTs were longer under water, see Fig. 5. In the isotonic condition, a significant and large effect was evident (${g} = 0.85$), and for the other haptic conditions, moderate-to-large-effect sizes and significant contrasts were found (except for stiffness 2, $p = 0.059$).

### Tracking

**Fig. 6:**
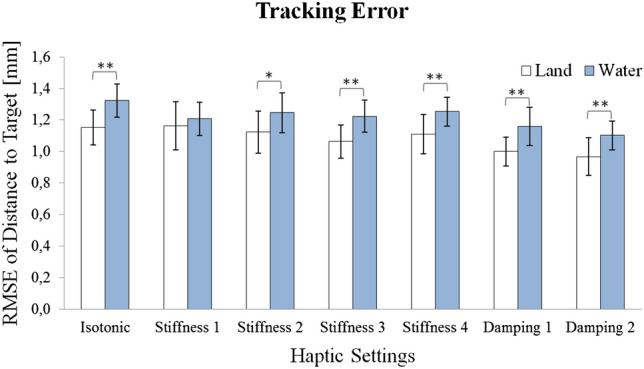
RMSE of tracking error for land (white) and water (blue) conditions with 95% confidence intervals. Significant differences are indicated by asterisk (*$p <0.05$; **$p <0.01$)

Tracking error increased significantly under water as reflected by a significant rmANOVA Setup main effect [$F \, (1, 19) = 17.06$; $p < 0.001$; see Fig. 6 and Table 3 for detailed statistical results]. Post hoc contrasts indicated significantly increased tracking errors with small-to-moderate-effect sizes in all haptic settings ($g = 0.67$ in the isotonic condition) except for stiffness 1. Moreover, a Haptic Setting main effect occurred [$F \, (6, 14) = 5.59$; $p <0.001]$. Tracking errors decreased significantly with damping 2 in both land and water conditions. A significant Direction main effect, $F \, (1, 19) = 43.52$; $p < 0.001$, also revealed tracking error anisotropy, i.e., the tracking error was higher on the horizontal compared to the vertical movement axis.

**Table 3:** Result overview for the tracking experiment with tracking error (mm)

${N} = 20$	Isotonic	Stiff. 1	Stiff. 2	Stiff. 3	Stiff. 4	Damp. 1	Damp. 2	rmANOVA results	
Land	1.153	1.162	1.123	1.063	1.111	1.000	**0.967**	**Setup:**	$\varvec{p} <\mathbf{0.001 }$
	(0.251)	(0.348)	(0.307)	(0.241)	(0.283)	(0.209)	(0.273)	F(1,19)= 17.60	($\eta$^2^ = 0.48)
*t*-Test (isot.-haptic)							$\varvec{p} <\mathbf{0.05 }$	**Haptic:**	$\varvec{p} <\mathbf{0.001 }$
*g* (isot.-haptic)		0.03	0.10	0.36	0.15	0.65	0.70	*F*(6, 14) = 5.59	($\eta$^2^ = 0.23)
Water	1.323	1.208	1.247	1.224	1.253	1.159	**1.102**	**Direction:**	$\varvec{p} <\mathbf{0.001 }$
	(0.243)	(0.242)	(0.289)	(0.234)	(0.210)	(0.277)	(0.207)	*F*(1, 19)= 43.52	($\eta$^2^ = 0.70)
*t*-Test (isot.-haptic)							$\varvec{p} =\mathbf{0.001 }$	**Direction** $\times$**Setup:**	$\varvec{p} <\mathbf{0.001 }$
* g* (isot.-haptic)		0.46	0.28	0.41	0.31	0.61	0.96	*F*(1, 19)= 27.39	($\eta$^2^ = 0.59)
*t*-Test (W-L)	$\varvec{p} < \mathbf{0.01 }$	n.s.	$\varvec{p} <\mathbf{0.05 }$	$\varvec{p} <\mathbf{0.01 }$	$\varvec{p} <\mathbf{0.01 }$	$\varvec{p} <\mathbf{0.01 }$	$\varvec{p} <\mathbf{0.01 }$	**Direction** $\times$**Haptic:**	$\varvec{p} < \mathbf{0.01}$
* g* (W-L)	**0.67**	0.14	**0.41**	**0.66**	**0.56**	**0.64**	**0.55**	*F*(3.40, 64.63)= 4.32	($\eta$^2^ = 0.19)
								Greenhouse–Geisser	

Means, standard deviations (in parentheses), significant results of rmANOVA (*p* values and partial ${{\eta }^{2}}$), effect sizes (Hedges’ *g*) and post-hoc contrasts for water–land (WL) comparisons and post hoc contrasts for the haptic setting comparisons (isotonic vs. haptic condition; significant means shown in bold)

Interestingly, this anisotropic effect was moderated by the setup [sign. Direction $\times$Setup interaction; $F \, (1, 19) = 27.39$; $p < 0.001$]: water immersion solely affected the vertical tracking error (land: ${\text {Error}}_{\text {horizontal}} = 1.256$ (0.259) mm; ${\text {Error}}_{\text {vertical}} = 0.910$ (0.206) mm; water: ${\text {Error}}_{\text {horizontal}} = 1.261$ (0.215) mm; ${\text {Error}}_{\text {vertical}} = 1.172$ (0.188) mm, ${g_{\text {W-L}}} = 1.30$). Finally, a significant Direction $\times$Haptic Setting interaction effect was found [$F \, (3.40, 64.63) = 4.32$; $p < 0.01$]. This was mainly due to a stronger positive effect of activating damping for the horizontal axis.

### Workload, training effects, and diving experience

#### Workload

Setup $\times$Haptic Setting rmANOVA on workload ratings did not reveal any significant effects.

#### Training effects

In the current experiment, there was only one initial training trial for both setups. Thus, potential training effects within the subtasks of one haptic setting could have had an effect. This was analyzed by an additional Subtask (aiming subtask or tracking subtasks in their chronological order) $\times$Setup (water vs. land) $\times$Haptic Setting rmANOVA. Significant main effects of Subtask were found for aiming RT and RMT. Specifically, the times for the first subtask were longer compared to the subsequent subtasks. However, this occurred in both setups and there were no significant Subtask $\times$Setup or Subtask $\times$Setup $\times$Haptic Setting interactions.

#### Diving experience

Additionally, we explored potential effects of diving experience by performing Direction $\times$Setup $\times$Haptic Setting repeated-measures ANCOVA with diving experience (number of dives) as covariate on all performance measures. Yet, we did not find any statistically relevant main or interaction effects of diving experience. The same applied to the workload ratings.

## Discussion

In the present study, shallow water immersion was used to investigate the effects of simulated weightlessness on sensorimotor performance during joystick controlled aiming and tracking tasks. Various haptic settings, i.e., spring stiffness and motion damping of the joystick, were compared. The study aimed to answer two main objectives: (A) the determination of preferable haptic settings of the HMI for simulated weightlessness and (B) a better understanding of the causal mechanisms triggering sensorimotor deficits in simulated weightlessness and how haptic settings potentially moderate these mechanisms.

### The effects of haptic settings in simulated weightlessness

In the present study, simulated weightlessness had the expected negative effect across all aiming and tracking performance measures. More surprisingly, however, results revealed that simulated weightlessness leads to increased response times only for certain haptic settings. A response slowing emerges in haptic settings with higher or continuous reaction forces (stiffnesses $\ge 0.375$ Nm/rad and both dampings). No effects are evident when no or low stiffnesses are activated.

The expected slowing effect of simulated weightlessness on rapid motion times also occurs during water immersion. Yet, divergent haptic setting patterns were evident in both setups. On land, rapid motion times linearly increase with stiffness and are increased with high damping (0.09 Nm s/rad). Obviously, subjects need more time moving against the respective counteracting forces. The pattern is slightly different under water: moderate stiffness (0.375 Nm/rad) as well as low damping (0.045 Nm s/rad) already produce significantly longer motion times compared to the land condition. Seemingly, rapid motions are additionally slowed down when relatively moderate haptic settings are applied in simulated weightlessness. Please note that these findings are also consistent with results of a current case study of the authors (Weber et al. 2019), showing that motion damping during a real telerobotic experiment is beneficial in terrestrial conditions but detrimental during spaceflight.

Regarding fine motion times, no substantial effects of haptic settings are evident. While haptics did not have any positive effect on precision aiming on land, there is at least initial evidence that there are haptic conditions exerting a small positive effect on fine matching times: low stiffness (0.075 Nm/rad) and high damping (0.09 Nm s/rad).

Damping also had the expected beneficial effect on tracking accuracy both on land as well as under water. Interestingly, this positive effect of providing motion damping of 0.09 Nm s/rad compared to the isotonic baseline condition is similar in both setups. Yet, the performance gap between both setups remains on a significant level with a moderate effect size. This result pattern indicates that there is no additional effect of simulated weightlessness on force regulation during slow, continuous motions. Thus, the same stabilizing mechanism of damping can be utilized in simulated weightlessness. It is also noteworthy that no significant weightlessness effect occurs for low stiffness (0.075 Nm/rad). While adding low stiffness had no effect on land, an almost moderate, positive effect size of $g = 0.46$ (although not reaching statistical significance) was evident under water. Stiffness mainly supports one aspect of the tracking task: motion reversals when reaching the turning points and thus maximal joystick deflection. Increasing the spring stiffness did not yield additional positive effects, i.e., very subtle haptic support is already sufficient in simulated weightlessness.

Altogether, the results emphasize that haptic support is indispensable for maintaining sensorimotor performance for high precision motions in simulated weightlessness. Here, motion damping should be enabled. For rapid motions, weaker haptic settings should be preferred in simulated weightlessness. Finally, the results also provide the initial evidence that low stiffness might be a good compromise for tasks including rapid as well as fine motions with frequent direction changes.

### Simulated weightlessness effects and potential explanations

The results show that feedforward-controlled motions as well as feedback-controlled motions are impaired by simulated weightlessness induced by water immersion. A response slowing was evident for higher resistive haptic settings. This raises the question, whether a cognitive impairment due to water immersion might have triggered the effect. Nitrogen narcosis is known to degrade cognitive function, but this effect occurs in depths beneath 15–30 m (Abraini 1997). Yet, response slowing during reaction tasks has been documented even for shallow water immersion in 5 m depth (Dalecki et al. 2012a) and have been attributed to the higher ambient pressure. In the current study, however, there was no general slowing of responses, they solely occurred when higher resistive forces were activated, and subjective ratings of workload also did not indicate a general impairment during water immersion. Here, it is more plausible that planning rapid motions against an external force field in a state of weightlessness is more complex and takes longer (Dixon 1985; Henry and Rogers 1960).

When executing the rapid aiming motions, water immersion led to longer motion times in the isotonic condition. While this finding is consistent with prior studies on aiming, not the weightlessness aspect, but water viscosity might have caused the slowing due to the relatively fast motions in the medium (Kerr 1978). For several reasons, however, this does not seem plausible here: (1) the hydrodynamic drag coefficient (${C}_{\text {D}} A$) for forearm motions in the sagittal plane should be substantially lower than for motions in the transverse plane. Yet, we did not find any anisotropic effect here. (2) Moreover, the hydrodynamic drag for the velocities in the current experiment is only about 10.5g during aiming motions in the transverse plane [estimated with a ${C}_{\text {D}}A$ value of 0.36, see Goldstein (1969), and an average arm speed of 2 cm/s, 4 cm/s at the hand and none at the elbow]. (3) A study with exactly the same aiming experiment conducted with three astronauts (Weber et al. (2018); 2 weeks in space) yielded a very similar rapid motion slowing effect of about 120 ms in the isotonic condition. Provided that the observed slowing is a result of simulated weightlessness, it is less likely that a changed control strategy to avoid high reaction forces on the body is the main trigger, since it would be plausible to expect anisotropies for rapid motions (Mechtcheriakov et al. 2002). Rapid motion times during water immersion already increase with comparably moderate haptic settings. Besides higher demands in terms of motion planning, executing these motions was additionally slowed down compared to the land conditions. This result is consistent with prior studies, showing that force regulation is impaired in simulated weightlessness due to disturbed proprioception (Dalecki and Bock 2014; Dalecki et al. 2012b).

With regard to the precise, fine motion matching, even stronger effects of water immersion were evident which emerged consistently in all haptic settings. In general, this pattern matches the expected effect pattern of distorted proprioception (Fisk et al. 1993). The more relevant proprioceptive information is, the larger the effects on motion times.

Additionally, finely tracking a slowly and constantly moving target also leads to substantial losses of precision in simulated weightlessness. Furthermore, the effects of simulated weightlessness for the tracking task reveal a strong anisotropy, i.e., motions in the sagittal plane (vertical axis in the experiment), were less accurate in water compared to land. Tracking the target in this plane involves wrist joint deviation (radial and ulnar deviation) as well as elbow and shoulder joint motion (flexion and extension), while motions in the transversal plane are mainly performed by rotating the forearm with the elbow as pivot (pronation and supination). Hence, the distorted proprioception approach again seems most plausible to explain the results of the current study. Given that changed control strategy or water viscosity would have been the triggers of weightlessness effects, rapid motions should have been mainly affected and, here, anisotropy should have emerged, since the inertial as well as the drag anisotropy in the two motion planes are larger during these fast motions. The reason why anisotropy was evident during tracking but not during fine matching might be that during aiming only few positional corrections were necessary, while, during tracking, numerous speed corrections in the corresponding axis of motion had to be performed resulting in a higher overall deviation.

In sum, the results of the current study investigating the effects of simulated weightlessness are consistent with prior research on effects of weightlessness induced by parabolic and space flight. The overall pattern of slowed aiming movements and less accurate tracking movements also emerged during weightlessness simulation under water. While no evidence was found for any significant impact of water viscosity, we gathered evidence in favor of the distorted proprioception explanation as several other studies exploring the effects of short-term exposition to microgravity (e.g., Bock 1998; Bock et al. 1992; Fisk et al. 1993; Manzey et al. 2000). The fact that we did not find any support for an altered control strategy might be due to the comparably small arm movements in the current aiming paradigm which probably did not produce sufficient inertia moment to de-stabilize the weightless body. Interestingly, a recent study provided the initial evidence for an altered control strategy in a very similar aiming task and experimental setup with the same way of body stabilization during spaceflight (Weber et al. 2019). In this study, a similar joystick was used, but movement amplitudes were twice the amplitudes of the present study ($16^\circ$ instead of $8^\circ$).

## Limitations

One potential limitation of the current study is the way how neutral buoyancy was exerted during water immersion. Here, weight belts were individually adjusted, i.e., neutral buoyancy was applied to the center of mass and not to the limbs. Macaluso et al. (2016) reported that arm pointing motions are slowed down with neutral buoyancy applied to the center of mass as well as applied to the body limbs by means of a special suit, but the effect is stronger with the latter method.

Another general limitation of simulated weightlessness during underwater exposure compared to microgravity is that the gravito-inertial force is still present and processed by the vestibular system. Lackner and DiZio (2000) emphasized that muscle spindle activity is also modulated by vestibular activity. Other studies investigating isometric force production successfully demonstrated that the changes of proprioception due to weightlessness can also be shown during water immersion [exaggerated peak and end forces (Dalecki 2013)]. However, the specific effects attributed to vestibular dysfunction (higher initial forces) could not be documented in the underwater condition. Both, the fact that limbs were not neutrally buoyant and the unchanged vestibular function might produce lower effects of water immersion compared to microgravity.

Although we did not find any effects of subjects’ diving experience, another limiting factor still is that subjects have comparably little time to adapt to water immersion, additionally making comparisons with spaceflight experiments with intensively trained astronauts (who usually have more to time to adapt to microgravity) more difficult.

## Conclusions

The human performance in basic sensorimotor tasks is substantially deteriorated by weightlessness. Consistent with research on microgravity during spaceflight, we found similar effects when performing aiming and tracking tasks during shallow water immersion. The present study provides evidence that planning complex multi-limb motions against resistive forces and the execution of highly accurate aiming and tracking motions are more demanding in simulated weightlessness. The overall result pattern indicates that distorted proprioception is the main reason for the sensorimotor deficit during motion execution. Haptic properties of the manipulandum which are supportive under standard land conditions (e.g., motion damping to stabilize tracking) should also be applied in weightlessness, although the effect of simulated weightlessness itself cannot be eliminated using haptics. Finally, resistive forces should be reduced to a minimum when rapid aimed motions have to be performed in simulated weightlessness. This knowledge is crucial for professional diving and potentially for future space missions, where haptic devices are, e.g., used for teleoperation tasks (e.g., Riecke et al. 2016). In a next step, we will validate the current findings during a real space mission and explore the benefit of haptic support in the different stages of adaptation.
